# Undergraduate education in palliative medicine in Germany: a longitudinal perspective on curricular and infrastructural development

**DOI:** 10.1186/s12909-015-0439-6

**Published:** 2015-09-17

**Authors:** Benjamin Ilse, Bernd Alt-Epping, Isabel Kiesewetter, Frank Elsner, Johanna Hildebrandt, Alexander Laske, Alexandra Scherg, Christine Schiessl, On behalf of the Working Group on Medical Education of the German Society of Palliative Medicine (AG Bildung der DGP)

**Affiliations:** 1 Department of Neurology, University Medical Center, Göttingen, Germany; 2 Department of Palliative Medicine, University Medical Center, Göttingen, Germany; 3 Department of Palliative Medicine, Munich University Hospital, Munich, Germany; 4 Department of Anesthesiology, Munich University Hospital, Munich, Germany; 5 Department of Palliative Medicine, Uniklinik RWTH Aachen University, Aachen, Germany; 6 Vestische Children’s Hospital, University of Witten/ Herdecke, Datteln, Germany; 7 Department of Anaesthesiology, University Medicine Greifswald, Greifswald, Germany; 8 Sana Arztpraxen Rügen, Bergen, Germany; 9 Interdisciplinary Centre for Palliative Medicine, University Hospital Dusseldorf, Dusseldorf, Germany; 10 Department of Anesthesiology, University of Erlangen-Nuremberg, Erlangen, Germany; 11 Algesiologikum - Pain Center, Munich, Germany

**Keywords:** Palliative medicine, Cross-sectional subject, Implementation process, German medical faculties, Undergraduate education

## Abstract

**Background:**

In 2009, palliative medicine became an integrated and compulsory part of undergraduate training in Germany by legislation. After a transitional period, all medical faculties were required to provide adequate teaching with an according examination and certification procedure. In parallel, we conducted bi-annual surveys on all medical faculties in Germany to examine for potential discrepancies between the implementation process and their intended consequences on teaching time and content.

**Methods:**

Four consecutive bi-annual surveys (2006, 2008, 2010, 2012) of all 36 medical faculties in Germany were performed, using purposively for this study developed questionnaires. Likert scales and closed questions were analyzed descriptively.

**Results:**

Medical Faculty response rate increased from 50 % in 2006 to 88.9 % in 2012. Teaching coordinators in palliative medicine primarily had an anesthesiology or internal medicine background. There was a noted increase over time of the involvement of specialized palliative care units (PCUs) as providing the setting for education. The number of faculties that were able to offer a complete 16 weeks of training in palliative medicine during the “final year” rose steadily. In addition, increased patient-centered teaching formats have been implemented over time. The faculties which offered innovative teaching formats with actors as patients (standardized patient interaction) increased, as did the total number of mandatory examinations. The number of faculties that provided compulsory teaching in a condensed manner within a single academic year increased sharply from 3 of 31 responding faculties in 2010 to 19 of 32 responding faculties in 2012.

**Conclusions:**

Until now, teaching conditions and structures in palliative medicine in Germany have proven to be extraordinarily heterogeneous. Although professorships (“Chairs”) in palliative medicine proved to be particularly beneficial and supportive in curricular and structural development, only a minority of faculties provide leading academic positions in palliative medicine.

## Background

There’s clearly a great need for improved access to palliative care worldwide. The European Association for Palliative Care (EAPC) stresses that training in palliative care in medical schools has a major impact on the future of palliative medicine (PM). “All healthcare professionals and workers should be able to provide appropriate palliative care and thus need to be trained to provide the highest possible standards of care in order to meet the challenging needs of patients and families, irrespective of diagnosis” [1, 2].

PM education is faced with the particular challenge to develop, not only knowledge and skills, but appropriate attitudes towards the issues of caring for patients who will die as a result of their incurable disease. Studies of the effect of current PM education on the development of skills and competencies illustrate that newly qualified doctors and medical students feel inadequately prepared and have limited confidence regarding their competencies in PM [3, 4].

On a national and international level medical schools and scientific communities have addressed this issue and developed curricula for undergraduate education in PM [5] The EAPC White Paper on palliative care education outlines 10 interdisciplinary and interprofessional core competencies relating to the most relevant principles of palliative care [1, 2].

In Germany, during the last two decades, funding programs of the German Cancer Aid (and, to a lesser extent, of private trusts) have catalyzed palliative medicine as a core academic subject within medicine [6]. This funding has forced the development of clinical structures at university hospitals and endowed professorships (currently 8 of 10 endowed chairs [7]) to implement teaching and research capabilities in this new field of medicine.

A turning point in PM education occurred in 2009 when Palliative Medicine became an integrated and compulsory part of undergraduate medical training by legislation. Importantly, every medical student has to take an exam in palliative medicine to get the license to practice medicine (AppOÄ 2002/07/03, last change 2009/07/31, § 27 and enclosure 15 to § 29 passage 3 phrase 2).

After a transitional period, all medical faculties must now provide “adequate” teaching with an according examination and certification procedure. Adequate teaching comprises of facilitating knowledge, skills and attitude development in the fields of symptom control, ethics and law, patient/family/non-clinical caregivers perspectives, and clinical communication, as postulated by the German and European Association for Palliative Care. The 36 medical faculties in Germany started this implementation process from very different positions, varying from completely established academically experienced departments at some faculties, to other sites with virtually no palliative medicine infrastructure [6].

Between 2006 and 2012, four consecutive surveys on all medical faculties in Germany were performed to monitor the development of education in palliative medicine. The first two surveys (2006/08) were conducted: before the new legislation established palliative medicine as a compulsory subject; one survey immediately after spreading the new requirements (2010); and the last one after implementation should have been accomplished widely, in order to fulfill obligatory examination prerequisites in 2012. The results of each distinct survey have been published independently in German medical journals [6, 8, 9]; here we present the longitudinal perspective on curricular and infrastructural development of undergraduate education in palliative medicine.

The aim of this study is to examine for potential discrepancies between the implementation process establishing palliative medicine as core training, and the effect upon teaching time and clinician development.

## Methods

The four consecutive bi-annual surveys (2006, 2008, 2010, 2012) on all 36 medical faculties in Germany were performed using a paper-based but electronically readable questionnaire that was sent to the responsible deanery or the contact person respectively. We developed the questionnaire purposively for this study. The first two surveys were conducted by the German Medical Students’ Association (Bundesvertretung der Medizinstudierenden in Deutschland e.V., bvmd). In 2010, different professional associations cooperated in the delivery of the survey (German Association for Palliative Medicine DGP, German Association for Haematology and Oncology DGHO, German Cancer Society DKG, and German Association for the Study of Pain DGSS). Via this network, and palliative medicine education workshops [10] we were able to establish contacts within the faculties of medicine who could appropriately and accurately provide data.

Likert scales and closed questions were analyzed descriptively. Questions regarding teaching facilities and structures were asked consistently; other items, e.g., those related to content and subjective perceptions of the persons responsible for teaching in palliative medicine, varied between the years.

Excel was used for data management and analysis. Data was analyzed descriptively. According to local standards, no formal approval of the local ethics authorities was requested.

## Results

The response rate from medical faculties increased from 50 % in 2006 to 88.9 % in 2012. Faculties with an established chair position in palliative medicine constantly showed a response rate of 100 % (Table 1).

**Table 1:** Surveys on undergraduate education in palliative medicine from 2006 to 2012

Year	2006		2008		2010		2012	
Item	n	%	n	%	N	%	n	%
Response rates of medical faculties (*n* = 36)	18	50.0	30	83.3	31	86.1	32	88.9
Chair in palliative medicine	5		4		8		8	
Teaching coordinators’ discipline								
- Anesthesiology	5	27.8	9	30.0	8	25.8	10	31.3
- internal medicine	7	38.9	9	30.0	13	41.9	10	31.3
- neurology	2	11.1	2	6.7	2	6.5	4	12.5
- general / family practice	0	0	4	13.3	3	9.7	3	9.4
- pediatrics	0	0	0	0	1	3.2	2	6.3
- neurosurgery	0	0	1	3.3	0	0	0	0
- psycho-social care	0	0	0	0	0	0	1	3.1
- psychosomatic medicine	0	0	0	0	1	3.2	1	3.1
- radiation therapy	1	5.6	1	3.3	1	3.2	1	3.1
- Dermatology	1	5.6	0	0	0	0	0	0
- medical history	1	5.6	1	3.3	0	0	0	0
- philosophy	0	0	1	3.3	0	0	0	0
- unknown	1	5.6	2	6.7	2	6.5	0	0
Organizational aspects; care structures that have been involved in undergraduate teaching								
- specialized palliative care units (PCUs)	5	27.8	12	40.0	24	77.4	23	71.9
- access to PCUs at academic teaching hospitals	10	55.6	21	70.0	18	58.1	12	37.5
- specialized palliative home care team^a^	–	–	–	–	13	41.9	20	62.5
Final year training								
- complete 16 weeks of final year training	2	11.1	5	16.7	7	22.6	8	25
- short-term rotation into palliative medicine	0	0	8	26.7	11	35.5	19	59.4
Teaching formats								
- lectures	3	16.7	5	16.7	15	48.4	30	93.8
- bedside-teaching^b^	–	–	–	–	12	38.7	15	46.9
- optional in-depth courses^c^	10	55.6	19	63.3	21	67.7	6	18.8
Teaching methods^b^								
- actors as patients	–	–	–	–	8	25.8	17	53.1
- e-learning	–	–	–	–	2	6.5	7	21.9
Examination								
- mandatory examination	5	27.8	3	10.0	–	–	–	–
- Multiple Choice^b^	–	–	–	–	10	32.3	27	84.4
- OSCE^b^	–	–	–	–	3	9.7	4	12.5
- oral examination^b^	–	–	–	–	2	6.5	6	18.8
- thesis^b^	–	–	–	–	1	3.2	5	15.6

Multiple answers were permitted; %/ response rates

^a^has been defined by legislation in Germany not earlier than in 2007

^b^not collected 2006/2008

^c^2010, in 13 faculties palliative medicine offered as mandatory subject and in in-depth courses, in 8 faculties only offered in in-depth courses

### Staff

Teaching coordinators initially responsible for the delivery of PM education primarily came from anesthesiology or internal medicine. In 2010 and 2012, the majority of teaching coordinators had been specifically trained in palliative medicine (Table 1).

Academic training such as “train the trainer” courses (13 of 32 in 2012), master courses in palliative care (5 of 32 in 2012) or master courses in medical education MME (4 of 32 in 2012) had rarely been undertaken by the responsible faculty members. Even in the 2010 survey, two teaching coordinators admitted to have no specific qualifications in palliative medicine at all.

### Organizational aspects

During the past 6 years there has been a growing involvement of specialized palliative care units (PCUs) as the main setting for palliative medical undergraduate education (Table 1). Moreover, in recent years, new formats of specialized palliative care were also included in undergraduate education, such as palliative medicine consultation or specialized palliative home care services.

### Final year training

In Germany, the “final year” consists of 3 × 16 weeks of practical training (“practical year”) in internal medicine, surgery, and another discipline of free choice. The number of faculties that were able to offer complete 16 weeks of final year training in palliative medicine rose steadily between 2006 and 2012, from two to eight. Accordingly, the number of faculties offering a short-term rotation into the field of palliative medicine within another final year attachment increased; zero to 19.

### Teaching formats

Initially, palliative medicine was largely taught by didactic lectures. Lately, more patient-centered teaching formats, e.g., bedside-teaching, have been implemented over the time. Simultaneously, the number of faculties offering voluntary courses decreased markedly over the years.

### Teaching methods

The number of faculties that included innovative teaching formats, for example using actors as patients (standardized patient interaction), increased from 8 of 31 in 2010 to 17 of 32 in 2012. Accordingly, e-learning opportunities increased from 2 of 31 faculties in 2010 to 7 faculties in 2012.

### Examination formats

There was an increase in the total number of mandatory examinations. The number of faculty sites using multiple choice examinations increased from 10 in 2010 to 27 in 2012. Additionally, there were more practical examinations, such as oral examinations (2 to 6), objective structured clinical examinations (OSCE; (3 to 4)) and thesis requirements (1 to 5).

### Volume and distribution of teaching hours

The number of faculties which provided compulsory teaching in a condensed manner within a single academic year increased sharply from 3 of 31 responding faculties in 2010 to 19 of 32 responding faculties in 2012. Traditionally, faculties spread palliative medicine contributions across several semesters, underlining its cross-sectional character with numerous connecting factors. The comparison of the total teaching volume proved to be not meaningful as total numbers of hours could assessed against multiple teaching formats (seminars/bedside-teaching versus plain lectures) that demand divergent efforts. In 2012, an average of 23.5 lessons (45 min each) was taught, ranging from 12 to 43 lessons (Table 2).

**Table 2:** Amount of teaching hours in palliative medicine 2010 and 2012 (faculties with compulsory curriculum, *n* = 11)

	Teaching hours	Lecture	Seminar	Bedside teaching	e- learning	Simulated patients	Total median
2010^a^	1	0	0	1	1	0	–
	2	4	2	0	0	3	
	3	1	1	0	0	0	
	4	1	1	0	0	0	
	<5	4	6	3	0	0	
	No or unknown	1	1	7	10	8	
2012^b^	(Range)	11.1 (2–23)	6.6 (0–24)	4.7 (0–30)	0.3 (0–3)	1.4 (0–4)	23

^a^One teaching hour equals 45 min

^b^One teaching hour equals 60 min

### Involvement of medical students in curricular development

The majority of faculties (18 of 31 in 2010; 19 of 32 in 2012) involved the respective medical students in the development of a palliative medicine curriculum.

## Discussion

These four bi-annual surveys document the development and implementation of undergraduate education in palliative medicine in Germany, fueled by the legislative which made teaching in palliative medicine compulsory in medical education. This in itself, is a remarkable development as in most other developed countries, teaching in palliative medicine is not a mandatory subject at medical school [11–14]. In Germany, a number of positive changes in educational and clinical infrastructure could be observed over time. These included the level of training amongst teachers and the use of more sophisticated and innovative teaching formats, e.g., a drama module [15].

The implementation of compulsory teaching corresponded with a sharp decrease in voluntary teaching formats that often provided a more in-depth and reflective approach to this topic. This may well be due to limited resources of the (mostly small) palliative care departments that are in charge of undergraduate education; it seems to be likely that those departments will not be able to provide both compulsory and voluntary teaching formats. Given that teaching in palliative medicine should not require only the transfer of cognitive knowledge or skills but also a caring attitude that enables the students to take a stance on the problems and dilemmas in end-of life care, this decrease of attitude-seeking course formats is regrettable [16]. Similarly, the number of faculties founding their examination procedure on multiple choice questions increased over time, owing to the large number of medical students undertaking this training. This may also be a limitation as multiple choice questions are more suitable to verify cognitive knowledge and facts, but are not appropriate to evaluate aspects of a caring attitude and skills [17].

A positive finding is that the spectrum of course placements developed to be very broad within the curriculum, underlining the multi-disciplinary character of this subject [18]. The median number of teaching hours increased from 2010 to 2012 and seems to be comparable to other countries now [19].

On the other hand, our surveys expose that there are still a few medical faculties in Germany that do not provide the required infrastructure, curricular scope or medical expertise that would be necessary to provide appropriate, authentic teaching in palliative medicine or even to comply with the described legal provisions. The overburdened curriculum could be another reason for the heterogeneity in teaching palliative medicine of the medical faculties [12]. While the involvement of medical students in curricular development was constantly high, the development of an evidence-based curriculum in undergraduate palliative care education is just beginning [5, 20–22].

Even beyond legal requirements, end-of-life care, with its medical, ethical, epidemiological and social implications, is increasingly appreciated by the general society, and to provide sufficient expertise and an empathetic attitude to approach patients with incurable and advanced disease has to be expected from the medical faculties. In general, medical students very much appreciate the teaching of in palliative care, and the development in Germany fits both the requirements of the general society and the needs of medical students [23–25].

This longitudinal series of surveys has several limitations. The response rate differed between the four surveys, and from year to year, the composition of participating faculties varied, limiting longitudinal comparability. Despite the high response rate, data of some faculties remained incomplete, leading to variable sums (n) of informative statements. Even if questions regarding teaching facilities and structures were asked consistently, other items, e.g., those related to content and subjective perceptions of the persons responsible for teaching in palliative medicine, varied between the years.

## Conclusions

These longitudinal views on the development of undergraduate education in palliative medicine in Germany highlight structural changes such as teaching staff, formats and methods from 2006 to 2012. On the one hand, the introduction of palliative medicine as a compulsory part of undergraduate training by legislation has led to a minimal standard of teaching at every university; on the other hand, it has promoted the implementation of resource-sparing teaching and examination formats. Whether lectures and multiple choice questions are the best teaching and examination formats to educate attitude and psychosocial contents to care for dying patients is questionable. Apart from a few faculties with excellent longitudinal education in palliative medicine, the structures are very diverse.

To face the discrepancy between the requirements for undergraduate education in palliative medicine and reality we suggest developing a national curriculum in palliative medicine [5]. Innovative teaching and examination formats have to be conceived and integrated into the regular education activities. The faculties with a chair in palliative medicine could have a leadership role in this process. Further, a tight exchange of experiences between the teaching staff from all faculties in Germany is needed. In addition, students’ and patients’ feedback about the “outcome” of palliative medical education should be considered. By this, the implementation process of undergraduate teaching in palliative medicine, the described solutions and improvements but even more the observed problems, in part depending on local infrastructure and leading to inacceptable heterogeneity in quality and quantity of undergraduate teaching, may well serve as a model for other countries who intend to implement palliative medicine as a mandatory subject in undergraduate education.

## Abbreviations

EAPC: European Association for Palliative Care
MME: Master degree in medical education
OSCE: Objective structured clinical examination
PCUs: Specialized palliative care units
PM: Palliative medicine
